# Structural insights into NMDA receptor pharmacology

**DOI:** 10.1042/BST20230122

**Published:** 2023-07-11

**Authors:** Changping Zhou, Nami Tajima

**Affiliations:** Department of Physiology and Biophysics, Case Western Reserve University, Cleveland, OH, U.S.A.

**Keywords:** glutamate receptor, neuropharmacology, structure

## Abstract

*N*-methyl-d-aspartate receptors (NMDARs) comprise a subfamily of ionotropic glutamate receptors that form heterotetrameric ligand-gated ion channels and play fundamental roles in neuronal processes such as synaptic signaling and plasticity. Given their critical roles in brain function and their therapeutic importance, enormous research efforts have been devoted to elucidating the structure and function of these receptors and developing novel therapeutics. Recent studies have resolved the structures of NMDARs in multiple functional states, and have revealed the detailed gating mechanism, which was found to be distinct from that of other ionotropic glutamate receptors. This review provides a brief overview of the recent progress in understanding the structures of NMDARs and the mechanisms underlying their function, focusing on subtype-specific, ligand-induced conformational dynamics.

## Introduction

l-Glutamate is the principal excitatory neurotransmitter in the mammalian brain and exerts its functions by binding to and activating glutamate receptors. Ionotropic glutamate receptors (iGluRs) play critical roles in neurotransmission and fundamental brain functions, including learning and memory. There are four subfamilies of iGluRs — *N*-methyl-d-aspartate receptors (NMDARs), α-amino-3-hydroxy-5-methyl-4-isoxazole propionic acid receptors (AMPARs), kainate receptors (KARs), and delta receptors — each with distinct pharmacological and physiological properties [1–5]. Seven NMDAR subunits (GluN1, GluN2A–D, and GluN3A/B) have been cloned to date. Functionally, the NMDARs are unique in that they require two agonists, glycine (or d-serine depending on the brain region) and glutamate, for activation [2]; they are regulated by voltage-dependent Mg^2+^ blockage [6,7]; they form cation channels with high Ca^2+^ permeability [8]; and have slower kinetics than other iGluRs [9–12]. Interestingly, recent studies have demonstrated that mild membrane stretch can substitute for neurotransmitters and also activate NMDARs [13]. Most native NMDARs in the central nervous system are composed of GluN1 and GluN2 subunits. NMDARs containing GluN3 subunits display strikingly different functional properties, that is, they have low permeability to Ca^2+^, are almost insensitive to Mg^2+^ block, and can be activated by glycine alone [14–16]. Thus, in this review, we primarily focus on the NMDARs containing GluN1 and GluN2 subunits.

NMDARs and AMPARs are co-expressed in many neurons [17,18], and the combination of these receptors expressed at postsynapses defines the shape of excitatory postsynaptic currents (EPSCs). AMPARs generate the large, early components of EPSC, while NMDARs mediate the slower late component of EPSCs. The NMDARs are permeable for Na^+^, K^+^, and Ca^2+^ ions. Indeed, the NMDAR-mediated Ca^2+^ influx is the primary trigger for the induction of synaptic plasticity [1,19]. Both the hyper- and hypofunctions of NMDARs have been associated with a variety of brain diseases, including Alzheimer's disease, Parkinson's disease, epilepsy, schizophrenia, autism spectrum disorder, and mood disorders [20,21]. Consequently, targeting NMDARs is considered to be an effective therapeutic strategy for these neurological diseases and disorders, and great efforts have been devoted to developing NMDAR-targeting therapeutic compounds [1,22].

**Figure 1.**
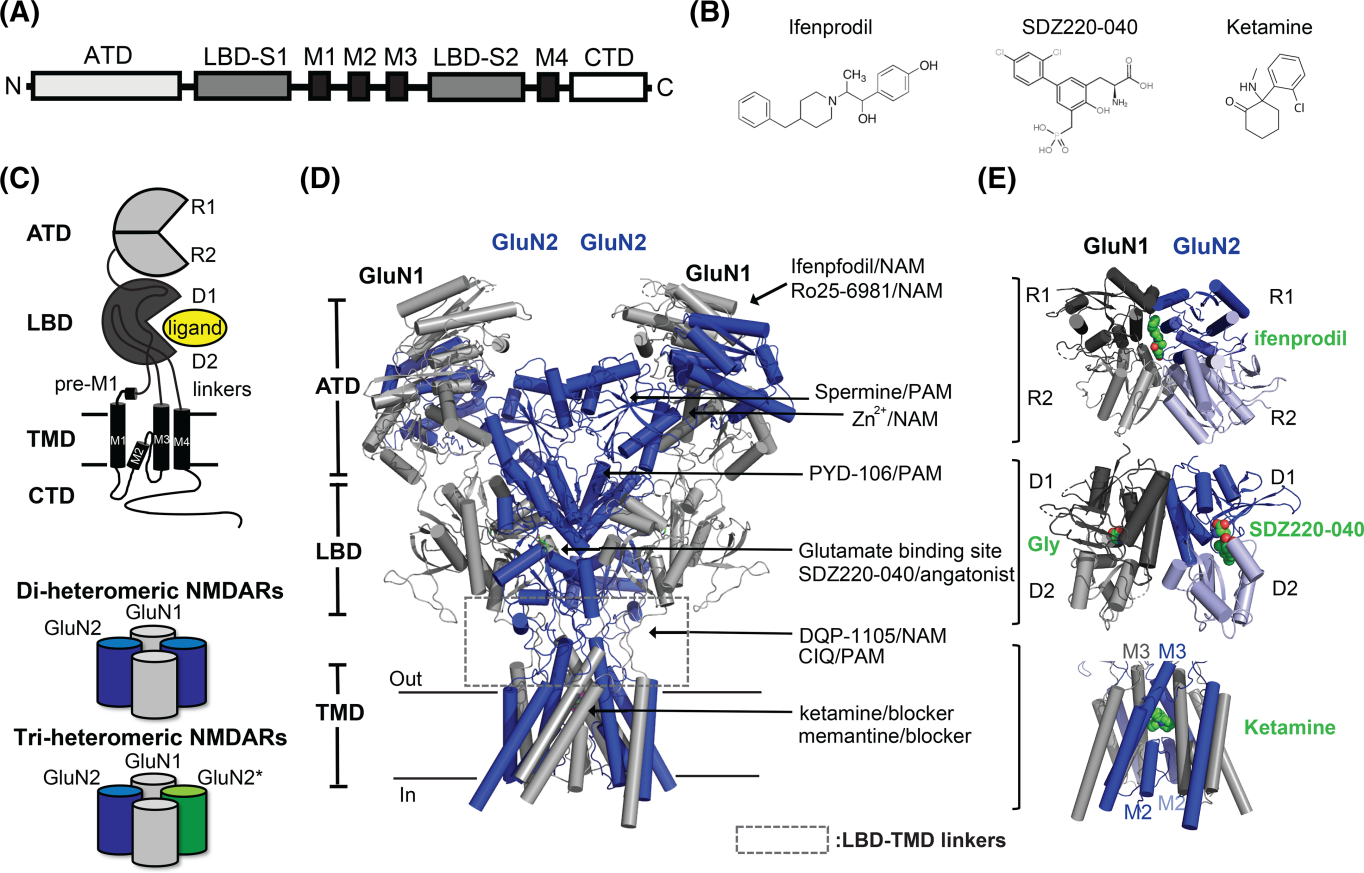
*N*-methyl-d-aspartate receptor (NMDAR) architecture and assembly. (**A**) Linear representation of the amino-terminal domain (ATD), ligand-binding domain (LBD), transmembrane domain (TMD), and C-terminal domain (CTD) within an NMDAR subunit polypeptide chain. The LBD comprises two separate polypeptides (S1 and S2) that fold into a clamshell-like structure. The TMD is formed by three transmembrane helices (M1, M3, and M4) and a membrane-reentrant loop (M2). (**B**) The chemical structures of the NMDAR ligands Ifenprodil (allosteric inhibitor), SDZ220-040 (competitive antagonist), and ketamine (open channel blocker). (**C**) Top: Common domain organization of NMDARs. The ATD and LBD are formed from two regions named R1 and R2 (ATD) and D1 and D2 (LBD). Agonists and antagonists docking between the LBD D1–D2 lobes are indicated (‘ligand’, yellow). The LBD and TMD are connected via three linkers, namely, S1–M1 (D2–M1), M3–S2 (D2–M3), and S2–M4 (D1–M4) (‘linkers’). Bottom: NMDAR subunit combinations. NMDARs are assembled as di-heteromeric or tri-heteromeric complexes. The latter contain two different GluN2 subunits (e.g. GluN1/GluN2A/GluN1/GluN2B). (**D**) Cryo-electron microscopy (Cryo-EM)-resolved structure of an intact di-heterotetrameric GluN1/GluN2B NMDAR in complex with S-(+)-ketamine, with the CTD deleted (PDB ID: 7SAC), with overviewing different NMDAR ligand-binding sites. The GluN1 and GluN2 subunits are in gray and blue, respectively. The LBD–TMD linkers are highlighted (gray box). (**E**) Ligand-binding sites. Top: Ifenprodil binding to the GluN1–GluN2B ATD heterodimer interface (PDB ID: 5EWJ); middle: glycine and SDZ220-040 binding to the ligand-binding sites on GluN1 and GluN2B, respectively (PDB ID: 6USV); bottom: ketamine binding in the ion channel pore formed by the M3 helices of GluN1 and GluN2B (PDB ID: 7SAC).

**Figure 2.**
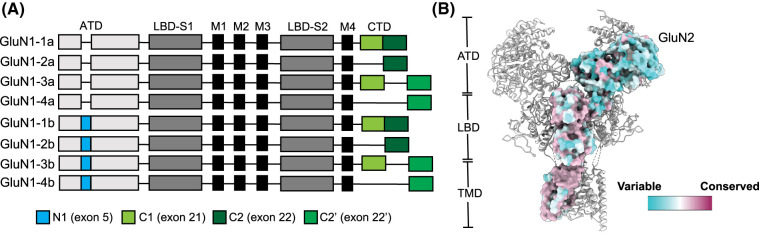
Structural conservation of surface-exposed amino acids in NMDARs. (**A**) Linear representation of the GluN1 polypeptide chain showing eight splice variants. The different GluN1 splice variants arise from the alternative splicing of exons 5, 21, and 22/22′, yielding the N1, C1, and C2/C2′ cassettes. (**B**) The conservation pattern of surface-exposed amino acids in human GluN2A–D subunits based on the structures of GluN1/GluN2A (PDB ID: 7EU7), GluN1/GluN2B (7EU8), GluN1/GluN2C (8E93), and GluN1/GluN2D (8E96). The color-coding bar shows the coloring scheme generated using Al2CO. Conserved amino acids are in purple, residues of average conservation are in white, and variable amino acids are in turquoise. The following NMDAR amino acid sequences were used: GluN1 (Uniprot # Q05586), GluN2A (Q12879), GluN2B (Q13224), GluN2C (Q14957), GluN2D (Q14957), GluN3A (Q8TCU5), and GluN3B (O60391).

The structures of the NMDARs are similar to, but distinct from, those of other iGluR families. iGluRs share a conserved domain organization comprising an amino-terminal domain (ATD), a ligand-binding domain (LBD), a transmembrane domain (TMD), and a carboxy-terminal domain (CTD) (Figure 1A) [1]. As mentioned above, there are multiple subtypes of NMDARs which differ in their subunit composition, and some ligands bind and modulate the receptor functions subfamily or subunit specifically (Figure 1B,D). NMDARs form obligate heterotetramers, whereas other types of iGluRs can form functional homomers as well as heteromers. Most NMDARs are composed of two glycine-binding GluN1 subunits and two glutamate binding GluN2 subunits, in an alternating GluN1–N2–N1–N2 subunit arrangement (Figure 1C). The GluN1 subunit exists as eight RNA splice variants (GluN1-1a/b to GluN1-4a/b) (Figure 2A), while there are four subunits of GluN2 (GluN2A–D) and two of GluN3 (GluN3A/B) [21]. The unconventional GluN1/GluN3-containing receptors, which have two copies of the obligate GluN1 subunit and two of the GluN3 subunit in place of GluN2, are only activated by glycine and generate excitatory conductance [23]; however, their roles are less well understood in comparison with GluN1/GluN2 NMDARs. Moreover, the NMDARs are highly diverse, forming di- or tri-heterotetramers (Figure 1C) [21]. The subunit composition of NMDARs determines their localization as well as their physiological, pharmacological, and signaling properties [21]. Furthermore, post-translational modifications, such as phosphorylation, ubiquitination, and palmitoylation, in addition to dynamic interactions with a wide range of proteins, further increase the diversity of NMDAR function and signaling [1,24].

Since the first crystal structure of an intact NMDARs was reported in 2014 [25,26], many intact NMDAR structures at resolutions of between 3.0 and 16.5 Å, mainly focused on the GluN2A and GluN2B containing receptors, have been determined by either crystallography or cryo-electron microscopy (cryo-EM) (Table 1) [25–37]. These intact receptor structures obtained in various functional states, together with previously determined crystal structures of isolated ATDs or LBDs, have provided detailed information regarding conformational changes in NMDARs. Combining insights from structural, physiological, and biophysical data with information gathered from molecular dynamics (MD) simulation analysis has led to the elucidation of the mechanisms involved in receptor activation, inhibition, and allosteric modulation. Here, we review recent developments in NMDAR structural pharmacology.

**Table 1.** Crystal and cryo-EM structures of intact NMDARs

Source	Functional state (conformation)	Ligand (conditions)	Resolution (Å)	PDB ID	Reference
**GluN1/2A**					
Human	Quasi-open	Glycine/glutamate	3.9	7EOS	[27]
Human	Non-active	Glycine/glutamate (pH 7.8)	4.5	6IRA	[28]
Rat	Pre-open/desensitized (2-knuckle)	Glycine/glutamate (+EDTA, pH7.4)	6.2	6MMG	[29]
Rat	Pre-open/desensitized (2-knuckle)	Glycine/glutamate (+EDTA, pH8.0)	6.9	6MMP	[29]
Human	Potentiated	Glycine/glutamate/GNE-6901	4.0	7EOR	[27]
Human	Antagonized	Glutamate/CGP-78608	3.8	7EOT	[27]
Human	Antagonized	Glycine/CPP	4.1	7EOQ	[27]
Human	Channel blocked	Glycine/glutamate/GNE-6901/9-AA	4.3	7EOU	[27]
Human	Channel blocked	Glycine/glutamate/S-ketamine	3.5 (4.5-6.0)*	7EU7	[30]
Human	Inhibited (class 1)	Glycine/glutamate/H^+^ (pH6.3)	5.1	6IRF	[28]
Human	Inhibited (class 2)	Glycine/glutamate/H^+^ (pH6.3)	5.5	6IRG	[28]
Human	Inhibited (class 3)	Glycine/glutamate/H^+^ (pH6.3)	7.8	6IRH	[28]
Rat	Inhibited (extended)	Glycine/glutamate/ZnCl_2_/H^+^ (pH6.1)	6.3	6MMA	[29]
Rat	Inhibited (super-splayed)	Glycine/glutamate/ZnCl_2_/H^+^ (pH6.1)	12.7	6MMB	[29]
Rat	Inhibited (1-knickle)	Glycine/glutamate/ZnCl_2_/H^+^ (pH6.1)	6.0	6MM9	[29]
Rat	Inhibited (extended-2)	Glycine/glutamate/ZnCl_2_/H^+^ (pH7.4)	8.2	6MMH	[29]
Rat	Inhibited (extended-1)	Glycine/glutamate/ZnCl_2_/H^+^ (pH7.4)	6.8	6MMM	[29]
Rat	Inhibited (splayed-open)	Glycine/glutamate/ZnCl_2_/H^+^ (pH7.4)	8.9	6MMI	[29]
Rat	Inhibited (super-splayed)	Glycine/glutamate/ZnCl_2_/H^+^ (pH7.4)	16.5	6MMJ	[29]
Rat	Inhibited (1-knuckle)	Glycine/glutamate/ZnCl_2_/H^+^ (pH7.4)	6.1	6MMK	[29]
Rat	Inhibited (2-knuckle, asymmetric)	Glycine/glutamate/ZnCl_2_/H^+^ (pH7.4)	7.1	6MML	[29]
Rat	Non-active (2-knuckle, symmetric)	Glycine/glutamate/ZnCl_2_/H^+^ (+EDTA, pH7.4)	5.1	6MMR	[29]
Rat	Non-active (2-knuckle, symmetric)	Glycine/glutamate/ZnCl_2_/H^+^ (pH8.0)	7.5	6MMN	[29]
**GluN1/GluN2A/GluN2A^H128S^**					
Rat	Non-active (2-knuckle, symmetric)	Glycine/glutamate/ZnCl_2_/H^+^ (+EDTA, pH7.4)	5.4	6MMS	[29]
Rat	Inhibited (1-knuckle)	Glycine/glutamate/ZnCl_2_/H^+^ (pH7.4)	7.5	6MMT	[29]
Rat	Inhibited (2-knuckle, asymmetric)	Glycine/glutamate/ZnCl_2_/H^+^ (pH7.4)	5.3	6MMU	[29]
Rat	Inhibited (2-knuckle, asymmetric)	Glycine/glutamate/ZnCl_2_/H^+^ (pH7.4)	4.7	6MMV	[29]
Rat	Non-active (2-knuckle, symmetric)	Glycine/glutamate/ZnCl_2_/H^+^ (pH7.4)	6.2	6MMW	[29]
Rat	Inhibited (extended)	Glycine/glutamate/ZnCl_2_/H^+^ (pH7.4)	7.0	6MMX	[29]
**GluN1/GluN2B**					
Rat	Active	Glycine/glutamate	6.8	5FXG	[31]
Rat	Active	Glycine/glutamate	4.4	6WHT	[32]
Rat	Active	Glycine/glutamate (cysteine cross-linking)	3.6	6WI1	[32]
Rat	active	glycine/glutamate/Fab5 antibody	7.5	7TEQ	[33]
Rat	Non-active 1	Glycine/glutamate	5.0	5FXH	[31]
Rat	Non-active 2	Glycine/glutamate	6.4	5FXI	[31]
Rat	Non-active 2 like (class x)	Glycine/glutamate	6.3	5FXJ	[31]
Rat	Non-active 2 like (calss Y)	Glycine/glutamate	6.4	5FXK	[31]
Frog	Non-active	Glycine/glutamate	7.0	5IOU	[34]
Rat	Non-active 1	Glycine/glutamate	4.0	6WHS	[32]
Rat	Non-active 2	Glycine/glutamate	4.0	6WHR	[32]
Rat	Non-active 1	Glycine/glutamate	3.0	7SAA	[35]
Rat	Non-active 1	Glycine/glutamate/Fab2 antibody	3.9	7TE9	[33]
Rat	Non-active 1 like	Glycine/glutamate/Fab2 antibody	4.2	7TEB	[33]
Rat	Non-active 2 like	Glycine/glutamate/Fab2 antibody	6.6	7TEE	[33]
Rat	Non-active 2	Glycine/glutamate/Fab5 antibody	5.2	7TER	[33]
Rat	Non-active 1	Glycine/glutamate/Fab5 antibody	4.7	7TES	[33]
Rat	Non-active 2 like	Glycine/glutamate/Fab5 antibody	4.5	7TET	[33]
Frog	Antagonized (class 1)	DCKA/D-APV	9.3	5IPV	[34]
Frog	Antagonized (class 2)	DCKA/D-APV	13.5	5IPQ	[34]
Frog	Antagonized (class 3)	DCKA/D-APV	14.1	5IPR	[34]
Frog	Antagonized (class 4)	DCKA/D-APV	13.5	5IPS	[34]
Frog	Antagonized (class 5)	DCKA/D-APV	14.1	5IPT	[34]
Frog	Antagonized (class 6)	DCKA/D-APV	15.4	5IPU	[34]
Rat	Antagonized (class 1’)	Glycine/SDZ220-040	4.1	6WHW	[32]
Rat	Antagonized (class 2’)	Glycine/SDZ220-040	4.1	6WHX	[32]
Rat	Antagonized (class 1”)	L689,560/glutamate	4.1	6WHY	[32]
Rat	Antagonized (class 2”)	L689,560/glutamate	4.3	6WI0	[32]
Rat	Antagonized (class 1”’)	SDZ220-040/L689,560	3.9	6WHU	[32]
Rat	Antagonized (class 1”’)	SDZ220-040/L689,560	4.1	6WHV	[32]
Rat	Inhibited	Glycine/glutamate/ifenprodil	4.0	4PE5	[25]
Frog	Inhibited/channel blocked (structure 1)	ACPC/t-ACBD, Ro25-6981/MK-801	3.6	4TLL	[26]
Frog	Inhibited/channel blocked (structure 2)	ACPC/t-ACBD, Ro25-6981/MK-801	3.8	4TLM	[26]
Frog	Inhibited	Glycine/glutamate/Ro25-6981	7.5	5IOV	[34]
Rat	Inhibited/channel blocked/GluK1-exon5 modulated	Glycine/glutamate/ifenprodil/MK-801	4.6	6CNA	[36]
Human	Channel blocked	Glycine/glutamate/S-ketamine	4.1 (∼3.8)*	7EU8	[30]
Rat	Channel blocked	Glycine/glutamate/S-ketamine	3.7 (∼3.0)*	7SAC	[35]
Rat	Channel blocked	Glycine/glutamate/phencyclidine	4.3 (3.5–4.0)*	7SAB	[35]
Rat	Channel blocked	Glycine/glutamate/memantine	4.0 (3.3–3.8)*	7SAD	[35]
**GluN1/GluN2C**					
Human	Pre-active (intact)	D-clycoserine/glutamate	4.0	8E92	[161]
Human	Non-active (splayed)	D-clycoserine/glutamate	3.7	8E93	[161]
Human	Pre-active (intact)	PYD-106	3.7	8E94	[161]
Human	Non-active (splayed)	PYD-106 (No PYD-106 bound)	4.2	8E97	[161]
Human	Pre-active (intact)	D-clycoserine/glutamate (nanodisc)	3.8	8E98	[161]
Rat	Non-active	Glycine/glutamate (major class)	3.6	7YFG	[162]
Rat	Potentiated	Glycine/glutamate/PYD-106	3.0	7YFH	[162]
Rat	Non-active	Glycine/glutamate (minor class)	4.3	8HDK	[162]
**GluN1/GluN2D**					
Human	Non-active	Glycine/glutamate	3.4	8E96	[161]
Human	Antagonized	Glycine/CPP	3.6	7YFF	[162]
Human	Non-active	Glycine/glutamate	3.9	7YFL	[162]
Human	GluK1-exon5 modulated	Glycine/glutamate	5.1	7YFM	[162]
Human	Super-active	Glycine/glutamate	6.4	7YFO	[162]
Human	Non-active	Glycine/glutamate	5.1	7YFR	[162]
**GluN1/GluN2A/GluN2B**					
Frog	Inhibited/channel blocked	Glycine/glutamate/Ro25-6981/MK-801	6.0	5UP2	[37]
Frog	Channel blocked	Glycine/glutamate/MK-801	4.5	5UOW	[37]
**GluN1/GluN2A/GluN2C**					
Human	Pre-active (intact)	D-clysoserine/glutamate/Nb4 nanobody	4.2	8E99	[161]
Rat	Non-active	Glycine/glutamate	3.3	7YFI	[162]

*: Local resolutions of TMDs where channel blockers bind.

## NMDAR architecture and function

### Amino-terminal domain

NMDAR ATDs allosterically modulate channel opening probability (Po), deactivation time course, agonist potency, and mean open-shut duration [38,39], and are also required for receptor assembly and trafficking [40–42]. ATDs show relatively low sequence identity (35–57% identify among the GluN2A–D subunits, and 22% identity between GluN1 and GluN3A) (Figure 2B), resulting in distinct functional and pharmacological properties among subunits [43]. NMDARs are unique in that some allosteric modulators bind to their ATDs and control channel activities which is not observed in AMPARs or KARs [43–45]. Over the past 20 years, numerous studies have shown that NMDAR ATDs adopt a clamshell-like conformation consisting of an upper (R1) and a lower (R2) lobe, which spontaneously oscillate between the open- and closed-cleft conformations (Figure 1C–E) [42,44,45]. The GluN1 and GluN2 ATDs form heterodimers through extensive interaction between the ATD R1–R1 interface (two heterodimers are arranged as dimer-of-dimers in the tetramer assembly) [43,46]. The ATD clamshell is an open conformation in the apo state, while some allosteric inhibitors induce the closure of the clamshell in the GluN2 ATD [1,28,39,43,47–49]. Extracellular polyamines also bind to the GluN1/GluN2B ATD interface and potentiate receptor function [36,50,51]. Similarly, some antibodies, such as autoantibodies associated with systemic lupus erythematosus, selectively bind to the GluN2A or GluN2B ATD and modulate NMDAR function [33,52].

### Ligand-binding domain

**Figure 3.**
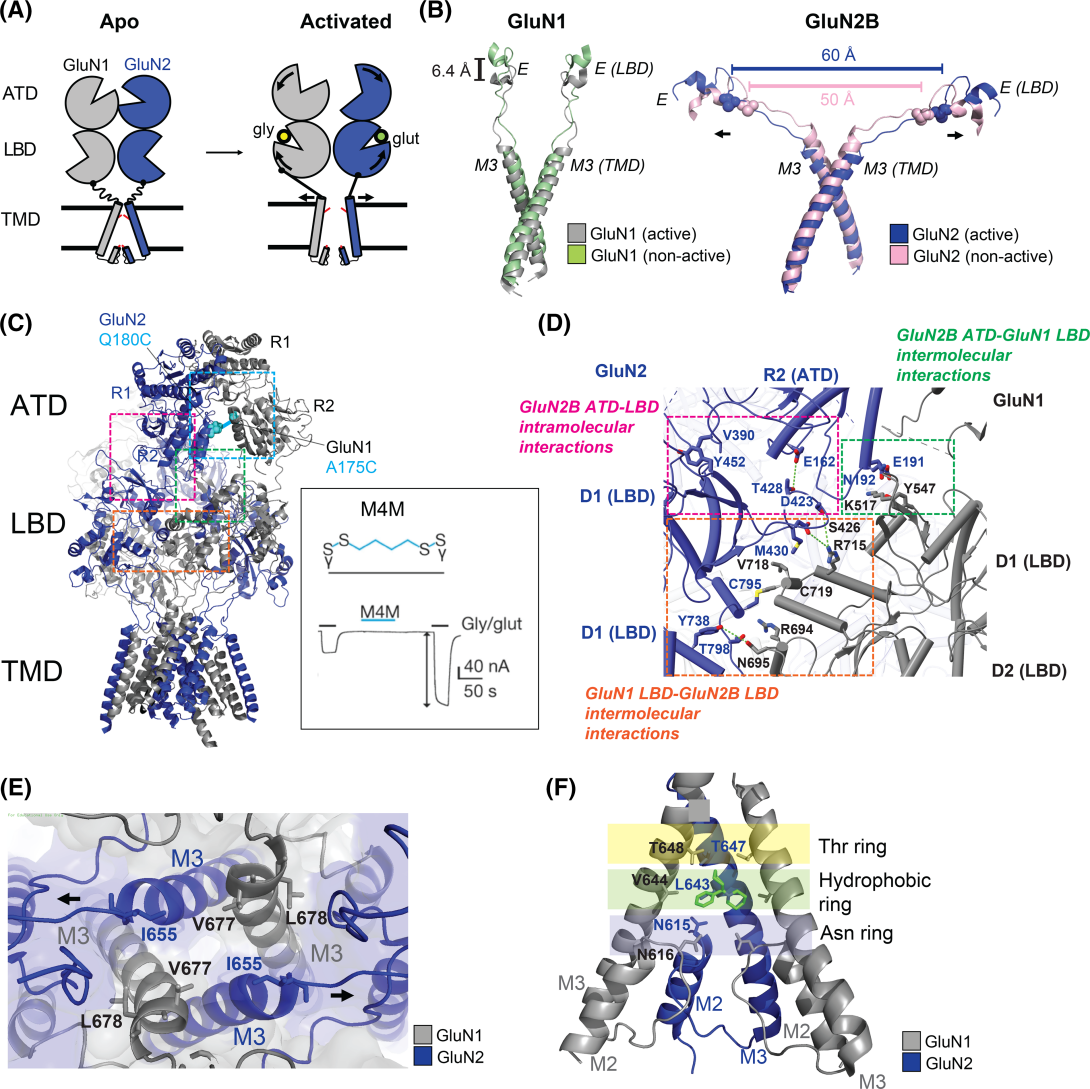
NMDAR activation and inhibition. (**A**) Schematic representation of NMDAR activation. Agonist binding at both GluN1 and GluN2 ligand-binding domains (LBDs) triggers clamshell closure. Clamshell closure generates tension in the LBD-M3 linkers and pulls the M3 helices, thus opening the pore. (**B**) Magnified views of the superposition of helix E in the LBD D2 lobe, pore-lining M3, and LBD-M3 linkers in GluN1 and GluN2B subunits in the agonist-bound ‘non-active 1’ (PDB ID: 6WHS) and the ‘active’ (PDB ID: 6WI1) structures. The spheres indicate the residue GluN2 (Q662) with cross-dimer distances. (**C**) Left: The structure of the GluN1/GluN2B NMDAR in the ‘active’ state highlighting the domain or subunit interfaces: ATD^GluN1^/ATD^GluN2^, ATD^GluN2^–LBD^GluN2^, ATD^GluN2^–LBD^GluN1^, and LBD^GluN1^–LBD^GluN2^ (cyan, pink, green, and orange, respectively). GluN1–GluN2B ATD heterodimer cysteine cross-linking (GluN1^A175C^/2B^Q180C^) is shown. Right: The application of a bifunctional cross-linker [1,4-butanediyl bismethanethiosulfonate (M4M)] to the GluN1^A175C^/2B^Q180C^ NMDAR mutant in the presence of glycine and glutamate potentiates the microscopic current as measured by two-electrode voltage clamp recording. Modified from Tajima et al. [31]. (**D**) Magnified view of the ATD–LBD and LBD–LBD interfaces in the GluN1/GluN2B NMDAR structure. (**E**) Extracellular view of the ion channel in an active GluN1/GluN2B NMDAR (PDB ID: 6WI1). (**F**) The channel blocker binding site and residues involved in the interaction. Modified from Chou et al. [35].

The LBD harbors the binding sites for agonists, competitive antagonists, and most allosteric modulators. GluN2 LBDs are highly conserved (69–82% sequence identity) (Figure 2B) [53] and display near-equal identity in the glutamate binding site. Both GluN1 and GluN2 LBDs also form clamshell-like structures containing an upper lobe (D1) and a lower lobe (D2), with ligands binding in the pocket between the two lobes (Figure 1C–E). GluN1/GluN2 LBDs form a back-to-back dimers via interactions between the D1 lobes (dimer-of-dimer arrangement in a tetramer) (Figure 1E). In the absence of ligands, the LBDs are in an open conformation. Agonist binding induces the bi-lobe closure, whereas the binding of an antagonist stabilizes the open conformation of the bi-lobe to a greater extent than that observed in apo-state structures [19,54–65]. Free energy calculations show that glutamate binds to the GluN2 subunits via a ‘guided-diffusion’ mechanism similar to that seen with other iGluRs; in contrast, glycine binds to the GluN1 and GluN3 subunits via an ‘unguided-diffusion’ mechanism whereby glycine finds its binding site primarily by random thermal fluctuations [66]. LBDs are tethered to the TMDs through flexible polypeptide linkers (Figure 1C,D). Therefore, signals resulting from clamshell conformational changes in the LBDs are transduced to the TMDs through these linkers (Figure 3).

### Transmembrane domain

**Figure 4.**
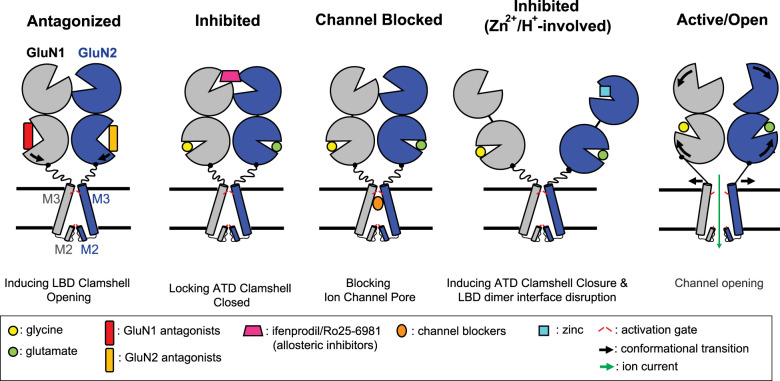
Schematic summary of NMDAR modulation by ligands and Ions. *Antagonism:* The binding of competitive antagonists causes the ligand-binding domain (LBD) bi-lobe to close, resulting in the closing of the ion channel pore. *Inhibition:* Some allosteric inhibitors such as ifenprodil and Ro25-6981 bind at the GluN1–GluN2 amino-terminal domain (ATD) heterodimer interface and close the ATD bi-lobe, blocking the receptors in inactive conformations. *Blocking:* Channel blockers inhibit NMDAR by occluding the ion channel. *Inhibition (Zn^2+^/H^+^-involvement):* Zn^2+^ and protons induce clamshell closure in the GluN2 ATD and close the ion channel. *Active/Open*: Glycine/glutamate binding triggers conformational changes in the LBDs of GluN1/GluN2 NMDARs. The conformational rearrangement of the GluN1–GluN2 ATD couples the GluN1–GluN2 LBD with downstream gating.

The TMD forms a cation channel. The NMDAR TMDs are highly conserved and share structural and functional similarities with potassium channels and other iGluRs [25,67]. The TMDs of iGluRs contain three membrane-spanning helices (M1, M3, and M4) and a membrane-reentrant pore-forming M2 loop (Figure 1A,C). The TMDs of NMDARs exhibit a quasi-four-fold symmetry whereases the ATDs and LBDs have two-fold symmetry. The M3 helices line the ion channel pore and form a primary activation gate on the extracellular side, whereas recently obtained evidence has indicated that the M2 loop of the GluN1 subunit acts as a second gate on the intracellular side [68]. The M1 and M4 helices surround the M3 helices forming the ion channel periphery. The tetrameric M3 bundle crossing forms a constriction point (or ‘activation gate’) that seals the ion permeation pathway in the closed state (Figure 4). Breaking the M3 bundle seal results in the opening of the ‘activation gate’ via an iris-like motion, which allows ion permeation. The bundle crossing of the M3 helices contains the SYTANLAAF motif that is highly conserved in iGluRs, implying that all iGluRs including NMDARs, share the basic gating mechanism. The cryo-EM structure of AMPARs in the open state [69] showed that M3 helices in the B/D subunits, which correspond to GluN2 subunits in NMDARs, are kinked at the alanine hinge in SYT**A**NLAAF when the gate is open. The structure of the ‘active’ state of NMDARs without auxiliary proteins does not show the kinked M3 helices. However, the mutagenesis [70] and AMPAR structure analysis have indicated that additional conformational changes in the channel gate might occur when NMDARs are activated, as seen with AMPARs [71,72]. In addition to M3, both pre-M1 and pre-M4 linkers are located near the SYTANLAAF motif, suggesting that they also participate in the channel gating. The distinct positions of these linkers could influence the durations of the intermediate conformations [73–76]. The N-terminal helical portion of the M2 loop forms extensive cross-subunit interfaces, while its C-terminal portion lines the intracellular side of the channel pore forming a selectivity filter that determines the Ca^2+^ permeability, single-channel conductance, and channel block (Figures 3F and 4) [2,77–84]. One of the unique functional features that distinguishes NMDARs from other iGluRs is that NMDARs have three- to four-fold higher Ca^2+^ permeability [2,21,84] and the permeability largely depends on the GluN2 subunits [85–88]. Analysis of the crystal structures of intact GluN1/GluN2B NMDARs showed that extracellular Ca^2+^ binds to the acidic DRPEER motif at the extracellular end of M3 in the GluN1 subunit, confirming that NMDARs have a charge-based calcium pooling mechanism similar to purinergic (P2X) and acetylcholine receptors [25,89,90]. Single-channel recordings have demonstrated that the external Ca^2+^ can block the receptors, indicating that there are additional Ca^2+^ binding sites in the ion channel pore [72,88,91].

### C-terminal domain

The CTD modulates the NMDAR channel activity, trafficking, and downstream signaling through the binding of intracellular ions, post-translational modifications, and alternative splicing [19]. The intracellular CTDs also contain binding sites for the synapse-associated proteins, including the postsynaptic density (PSD) family of membrane-associated guanylate kinases (PSD-MAGUKs) [92–95], Ca^2+^/calmodulin-dependent protein kinase II (CaMKII) [96–99], and synaptonuclear protein messengers [100–102]. The NMDAR CTDs are highly variable in sequence and length and are primarily disordered [103,104]. GluN2 subunits, in particular, have uniquely long CTDs (GluN1: ∼50 residues, GluN2: 396–696 residues, and GluN3: ∼200 residues) [105] relative to AMPARs and KARs (∼80 residues) and sequence identity is low among NMDAR subunits. Accordingly, the interactions with signaling/scaffold proteins depend on the subunit composition [106,107], and each subunit has distinct signaling properties [108,109]. In mice, GluN1 or GluN2B CTD homozygous deletion is perinatally lethal, while loss of the GluN2A or GluN2C CTD results in motor coordination deficits; mice lacking only the CTD of GluN2A have impaired memory [110,111]. Owing to the flexibility of the CTDs, all NMDAR structures obtained to date have been generated from CTD-deleted constructs. Therefore, no CTD structures are available, and how variations in the CTD affect the receptor functions remains unclear.

### Architecture of the intact heterotetrameric NMDAR

The first crystal structures of intact GluN1/GluN2B NMDARs in complex with the allosteric inhibitors ifenprodil or Ro25-6981 showed that NMDARs have a unique domain organization [25,26]. Like AMPARs, NMDARs display dimer-of dimers organization of the ATD and LBD, with domain swapping between the ATD and LBD layers. AMPARs and KARs have overall ‘Y’ shape and there is limited or no interaction between the ATD and LBD layers. In contrast, NMDARs exhibit ‘hot-air balloon-like’ compact conformations, with extensive interactions occurring at the ATD–LBD intramolecular interface in each of the four subunits as well as at the interface between the ATD of one subunit and the LBD of the adjacent subunit (Figure 3C,D). Therefore, conformational changes in the ATD layer of NMDARs are tightly associated with the LBD and TMD layers unlike non-NMDARs as mentioned above [31,34]. These structural features support a role for NMDAR ATDs in controlling receptor functions, including channel maximal open probability (Po), deactivation kinetics, activation, and allosteric inhibition [38,39,41,42].

NMDARs with different subunit combinations, such as di-heteromeric and tri-heteromeric receptors, also show distinct assemblies. The GluN1/GluN2A NMDARs exhibits more compressed dimeric ATD interactions and distinct inter-subunit LBD interactions compared with GluN1/GluN2B receptors [27–29]. Together with the electrophysiological data, these characteristics may provide a structural explanation for the subunit-selective allosteric modulation, and may also explain why the deactivation/desensitization kinetics of GluN1/GluN2A are faster than those of GluN1/GluN2B receptors [37,112]. The structure of the tri-heteromeric GluN1/GluN2A/GluN2B NMDAR, the major subtype expressed in the hippocampus and cortex, shows a unique subunit arrangement and symmetry, as well as distinct inter-subunit interactions, which are not observed in the di-heteromeric GluN1/GluN2A and GluN1/GluN2B structures [37]. The GluN1–GluN2A and GluN1–GluN2B heterodimers form a dimer-of-dimer arrangement in their tri-heteromeric assembly, thus displaying conformational asymmetry. Compared with that seen with the GluN1/GluN2B heterodimer side, the GluN1–GluN2A ATD heterodimer displays more extensive interactions, and the ATD and LBD of GluN2A are in closer contact, with stronger intramolecular interactions. The GluN1–GluN2A LBD heterodimer also forms substantial intermolecular interactions. These conformational arrangements increase the functional diversity of NMDARs and explain the complexity of NMDAR signaling.

### Gating mechanism of NMDARs

iGluRs share a common basic gating mechanism and their gating cycle can be categorized into four states, including (i) an agonist-free, closed-channel (resting) state; (ii) an agonist-bound, closed-channel (inactive) state; (iii) an agonist-bound, open channel (active) state; and (iv) an agonist-bound, closed-channel (desensitized) state (single-channel recordings indeed show complexity with multiple kinetically distinct states) [113]. During the transition from a resting to an active state, the agonist binding to the LBDs closes the LBD bi-lobes which pulls the LBD–TMD linkers, resulting in the opening of the ion channel pore similar to other iGluRs (see the *Agonist* section for a detailed description of the conformational alternations) (Figure 3A) [31,69,114–116]. However, comparative analysis between NMDARs and AMPARs in these functional states revealed distinctive assemblies and activation mechanisms. First, in NMDARs, a subtype-specific rolling motion of the ATDs allosterically controls the channel activities through the ATD–LBD inter-domain interactions [117,118] (Figure 3C,D). Secondly, NMDARs are unique in requiring two agonists for activation, and the LBD–LBD inter-subunit interactions are critical for this process. Unsurprisingly, the binding of glycine to GluN1 interferes with the glutamate binding to GluN2 subunits and vice versa, and the binding of one agonist allosterically modulates the binding of the second through the dimer–dimer interface which is not observed in non-NMDARs [119]. NMDARs exhibit only weak desensitization. Thus, the transition from an active to a desensitized states does not induce significant LBD rearrangement as happens with other iGluRs [80,120–122]. MD simulation analysis showed that the Arg–Glu salt bridge in NMDAR LBDs contributes to the receptor desensitization [123]. In summary, the gating mechanisms and kinetics of NMDARs are more complex than those of AMPARs. Ligand binding-induced conformational changes in LBDs as well as the subunit rearrangement and domain reorientation control the NMDAR gating. To understand the conformational changes occurring upon receptor activation and allosteric modulation in detail, the structures of intact heterotetrameric NMDARs in distinct functional states were recently determined, mainly by single-particle cryo-EM (Table 1). In the following section, we discuss how the conformational changes of each domain alter the receptor function.

## Structural pharmacology of NMDARs

### Agonists

The NMDARs are the most stringently gated iGluRs, requiring the simultaneous binding of glycine and glutamate in addition to membrane depolarization (release of the Mg^2+^ block) for activation. Analysis of the cryo-EM structures of intact NMDARs bound to glycine and glutamate showed that multiple conformational changes occur in the tetrameric assembly upon activation [27,31,32,34,117]. A comparison of the structures of NMDAR in the resting, agonist-bound non-active, and active states revealed that all three layers undergo a concerted conformational rearrangement (Figure 3A). Like other iGluRs, the LBD clamshell closure induced by the agonist binding moves the D2 lobe away from the membrane. Because D2 lobes are connected to the M3 helices via short linkers, D2 lobe reorientation pulls on the M3 helices, leading to the opening of the ion channel pore (Figure 3B–E). The earlier real-time, single-molecule fluorescence resonance energy transfer (smFRET) study showed that the glutamate binding to GluN2 triggers two sequential steps of GluN1–GluN2B ATD heterodimer separation, while the glycine binding to GluN1 allows GluN1 ATD rotation [124]. Indeed, the recent structures of intact GluN1/GluN2B NMDARs in the activated state showed these GluN1 ATD and GluN2B ATD rotations in addition to the GluN2 ATD clamshell opening. In contrast with non-NMDARs, NMDAR heteromerization involves extensive interactions between ATDs and LBDs, and the determination of the structures of NMDARs revealed how ATD controls channel gating [19,71,125]. When NMDARs are activated, a GluN1–GluN2 ATD ‘rolling down’ motion is translated into a ‘rolling up’ motion of LBD heterodimers. In addition to the LBD clamshell closure induced by agonists, this rolling-up motion pulls the LBD–TMD linkers, which moves the M3 helices, thereby opening the ion channel. These ATD and LBD rotations act as gating switches and represent an activation mechanism that is unique to NMDARs. Stabilizing the ‘ATD rolled down’ or ‘LBD rolled up’ intermediate state increases the receptor current, indicating that these rolling motions of both extracellular domains are critical for the NMDAR activation (Figure 3C) [31,117]. Because the extracellular domain of NMDARs has 2-fold symmetry, the movements of the GluN1 and GluN2 subunits differ. The D2–M3 linkers in the GluN1 subunits are perpendicular to the membrane while those in the GluN2 subunits are parallel (Figure 3B). Upon receptor activation, the GluN2 linkers extend out perpendicularly, opening the channel pore; the GluN1 linkers are not as strongly coupled to channel gating as those in the GluN2 subunits. Single-channel recording data have indicated that multiple, short-lived transition states exist between the agonist-bound pore-closed and pore-open states [73,126–128]. These intermediate states exist on a millisecond scale and have not been captured to date owing to technical difficulties associated with the use of cryo-EM or crystallography. However, MD simulation and double electron–electron resonance (DEER) data have confirmed the unique subunit-specific conformational alternations that occur in NMDARs upon activation and inhibition (e.g. linker movement or residue reorientations) [34,129,130].

### Allosteric inhibitors

**Figure 5.**
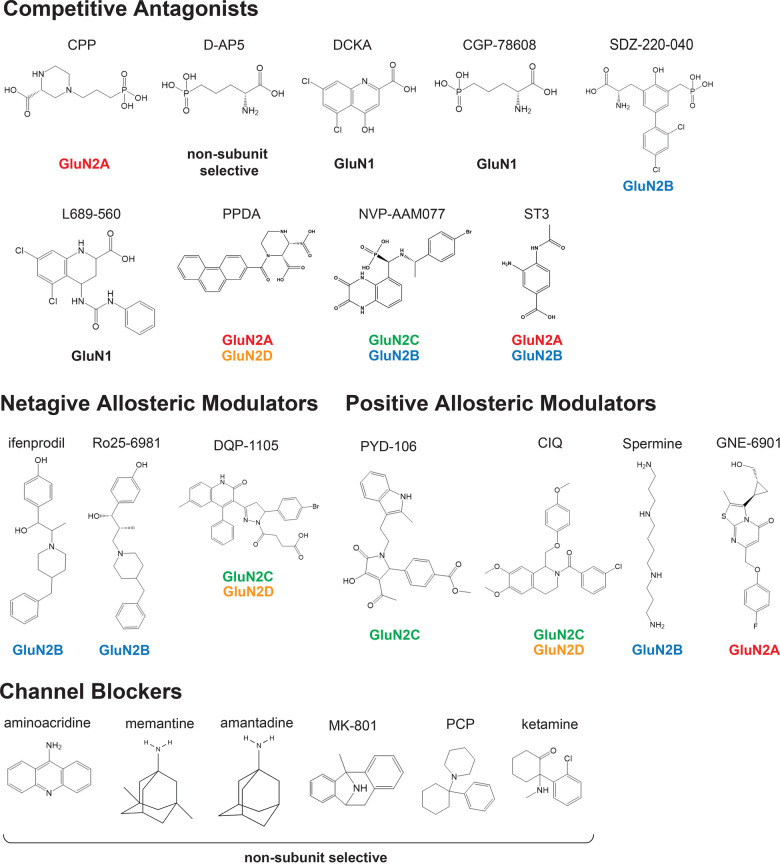
Chemical structures of antagonists, open channel blockers, and allosteric modulators of NMDARs. The chemical structures and the subtype specificity are shown.

Some allosteric modulators target the ATD and selectively inhibit NMDAR subtypes (Figure 5). Ifenprodil is a selective GluN2B inhibitor, displaying ∼400-fold greater selectivity for GluN2B than for other NMDAR subunits [131] (notably, ifenprodil has off-target effects; it also inhibits other receptors, including the adrenergic, serotonin, and sigma receptors that may cause adverse effects [132,133]). Analysis of the crystal structures of isolated GluN1–GluN2A and GluN1–GluN2B ATDs has shown that they have distinct conformations, which can explain subunit selectivity [43,45]. In addition, multiple crystal structures of the GluN1/GluN2B ATD complexed with GluN2B-selective allosteric inhibitors (e.g. ifenprodil, EVT101, and E93-31) have shown that all these inhibitors bind to the GluN1–GluN2B ATD heterodimer interface and stabilize the GluN2B ATD in an inactive, clamshell-closed conformation (Figure 1E), although the binding modes differ [25,26,45,134,135]. The structures of intact GluN1/GluN2B receptors bound to ifenprodil or Ro25-6981 allowed the elucidation of the mechanism underlying allosteric inhibition. For instance, ifenprodil induces the closure of the GluN2B ATD clamshell by ∼22° and the rotation of the GluN1–GluN2B ATD heterodimer by 12° relative to the active-state conformation. This ATD reorientation stabilizes the LBDs in inactive conformations, which promotes the closure of the ion channel gate (Figure 4). The critical residues in GluN2B involved in ifenprodil binding are not conserved in GluN2C/D or GluN3A/B, but are conserved in GluN2A and GluN2B. A recent study confirmed the uniqueness of subunit–subunit interfaces and subunit domain rearrangements, which explains how ifenprodil selectively inhibits GluN1/GluN2B but not GluN1/GluN2A NMDARs [118]. In contrast with the notable inhibition efficacy of ifenprodil against the di-heteromeric GluN1/GluN2B receptors (IC_50_ 72 ± 8 nM), ifenprodil only moderately inhibits tri-heteromeric GluN1/GluN2A/GluN2B (IC_50_ 450 ± 30 nM, with a maximal inhibition of 32 ± 1%) [136]; this indicates that inactivation of one subunit in the tetramer is not sufficient to fully block the receptor function [136–138].

Extracellular Zn^2+^ released into the synaptic cleft from presynaptic terminals along with glutamate also allosterically inhibits GluN2A-containing NMDARs in a proton-dependent manner. The resolution of the structure of the Zn^2+^-bound GluN1/GluN2A NMDAR showed that Zn^2+^ binds within the cleft of the GluN2A ATD clamshell and interacts with four residues, resulting in high binding affinity, but only interacts with two residues in GluN2B (GluN1/GluN2A IC_50_ = 0.02 μM, GluN1/GluN2B IC_50_ = 2.5 μM, GluN1/GluN2A/GluN2B IC_50_ = 0.058 μM) [29,43,139–142]. Like with ifenprodil, Zn^2+^ binding also facilitates the GluN2A ATD clamshell closure [43,143] by disrupting the D1–D1 interfaces of the LBD dimer, relieving the tension of the LBD-M3 linker, and stabilizing the closed-pore conformation in the intact GluN1/GluN2A structure [29]. Interestingly, in one of the Zn^2+^-bound receptor structure, the receptor was reported to adopt a ‘super-splayed’ conformation, with the complete separation of the ATD and LBD heterodimer pairs (Figure 3). This conformation may represent the low open probability (Po) state in the presence of a micromolar concentration of Zn^2+^ concentrations at low pH (pH 6.1). In contrast, the structures of the GluN1/GluN2A receptor structures without Zn^2+^ at higher pH (pH 7.4–8.0) may represent high Po states.

Finally, the recently resolved structure of the GluN1/GluN2B NMDAR bound to an inhibitory antibody showed that the antibody induces the closure of the GluN2B ATD clamshell via a long-range network, which stabilizes the inhibited stated, equivalent to a ‘non-active’ state of the intact receptor [33]. Unlike small compounds, this antibody reagent had no off-target effects, showing some potential promise as a subtype-selective reagent.

### Competitive antagonists

Competitive antagonists directly bind to the ligand-binding sites on the GluN1 or GluN2 subunits and inhibit the channel activity. Developing GluN2 subtype selective competitive antagonists may be challenging because the glutamate binding sites are highly conserved. Consequently, allosteric modulators are currently mainly used as pharmacological tools to distinguish the GluN2 subunits. Early crystal structures of ligand-bound, isolated LBDs combined with FRET analysis revealed that competitive antagonists of GluN1 and GluN2 occupied the glycine (GluN1) and glutamate (GluN2) binding sites, respectively, and stabilized the open LBD clamshell conformation to varying degrees (Figure 4) [57–59,62,144–146]. For example, the GluN2A LBD bi-lobes are opened ∼20°, 16°, and 13° in response to (−)-PPDA, *D*-APV/NVP-AAM007, and ST3, respectively, compared with glutamate-bound LBD [62,140].

The recently resolved cryo-EM structure of intact NMDARs in complex with antagonists explained the mechanisms underlying differential inhibition by GluN1 and GluN2 antagonists. Analysis of the intact GluN1/GluN2B NMDAR structures showed that the antagonist L689,560 (L689) opens the GluN1 LBD clamshell by ∼28° and SDZ-220-040 (SDZ, *D*-APV analogs) the GluN2B LBD by ∼23°, respectively compared with the glycine- or glutamate-bound structures like that seen in the crystal structures [32]. The LBD clamshell opening reduces the tension on the LBD–TMD linkers and induces the ion channel closure (Figure 4). This mechanism of antagonism is conserved in other iGluRs. However, antagonizing either the GluN1 or GluN2 subunit completely inhibits NMDAR channel function, which is not observed in AMPARs and KARs. A comparison of four structures, GluN1^gly^/GluN2B^glut^, GluN1^gly^/GluN2B^SDZ^, GluN1^L689^/GluN2B^glut^, and GluN1^L689^/GluN2B^SDZ^, revealed the mechanisms involved in subtype-specific inhibition [32]. The GluN1^L689^/GluN2B^glut^ structure is similar to the non-active/pre-open conformation, with L689 stabilizing the LBD clamshell in the open conformation, which relaxes the LBD–TMD linkers and closes the ion channel pore. In contrast, the structure of SDZ-bound GluN2B is more like that seen in the active state (i.e. ATD rolling down, and LBD clamshell rolling up), except that the linker is relaxed and the channel is closed. In the GluN1^L689^/GluN2B^SDZ^ structure, there are additional interactions at the top of the gate, and the ion channel pore is even more tightly closed in this structure than in the GluN1^L689^/GluN2B^glut^ and GluN1^L689^/GluN2B^SDZ^ structures.

### Open channel blockers

Owing their clinical importance, the NMDAR channel blockers have been investigated for decades. Open channel blockers (Figure 5) [40,147–150] initially bind deep within the pores of NMDARs, and only when the channel is open [151–153]. They are classified into the following three classes based on their interactions with pore-lining residues: (1) ‘sequential’ blockers, such as aminoacridine, which block open NMDAR channel, prevent channel closure and desensitization; (2) ‘partial trapping’ blockers, such as memantine and amantadine, which obstruct channel closure but cannot completely prevent it; and (3) ‘trapping’ blockers, such as MK-801, phencyclidine (PCP), and ketamine, which are trapped inside the pore as the channel closes, and allow agonist unbinding while the trapping blocker remains bound [19]. Indeed, memantine has been used for treating moderate to severe Alzheimer's disease and is in clinical trials for the treatment of epilepsy and schizophrenia [154,155], while ketamine is used for the treatment of depression [147,156]. In general, these channel blockers are non-NMDAR subunit selective. The recent elucidation of cryo-EM structures at high resolution combined with MD simulation has also demonstrated the precise binding modes for memantine, ketamine, and PCP, as well as the detailed channel gating dynamics [35]. Another cryo-EM-based study also showed that ketamine bound to similar residues in the GluN1/GluN2A and GluN1/GluN2B receptors [30]. The binding site comprises three layers, namely, the Thr, hydrophobic, and Asn gating rings, which control the dissociation speeds (Figure 3F) [35]. The association of these channel blockers with neighboring residues is mainly mediated by hydrophobic interactions. The MD simulations demonstrated that the channel blockers fluctuate within the binding site mainly in the plane parallel to the membrane, with ketamine displaying greater fluctuation in binding poses than PCP or memantine [35].

## Conclusion

Recent progress in the structural biology of NMDARs has significantly increased our understanding of the regulatory mechanisms by which ligand binding induces conformational changes and domain rearrangements in all layers of the receptors. These data have demonstrated the differences and commonalities between NMDARs and non-NMDARs. While there was tremendous progress, much more remains to be identified. For instance, understanding the structures of NMDARs carrying disease-associated mutations [157], and GluN1/GluN3 receptors that function as glycine receptors [158] will provide a better understanding of NMDAR subfamilies. Capturing the short-lived functional intermediate states using a time-resolved cryo-EM technique and a microfluidic chips [159,160] combined with MD simulation, FRET, and electron paramagnetic resonance (EPR) analysis is useful for unveiling these conformational transitions. Understanding how post-translational modifications and native lipids modify NMDAR structure and function is also essential. Various scaffolding and signaling proteins, including calmodulin–Ca^2+^/calmodulin-dependent kinase II (CAMKII) complexes and PSD-95, interact with NMDARs, transmit glutamatergic signals and induce synaptic plasticity. Thus, visualizing NMDAR–cofactor interactions is critical for understanding NMDAR subtype-unique signaling pathways [1]. It is hoped that structural information will promote and even accelerate the development of therapeutic agents for the treatment of NMDAR-associated brain diseases.

## Perspectives

- *N*-methyl-d-aspartate receptors (NMDARs) play a crucial role in synaptic plasticity, learning, and memory formation. NMDAR dysfunction is associated with a wide range of neurological and psychiatric disorders.
- Recent structural studies of NMDARs have provided valuable insights into their mechanism of activation, allosteric modulation, and inhibition. The unique assembly of NMDARs contributes to their distinct regulation mechanism compared with non-NMDARs.
- Despite the recent significant progress, unconventional NMDARs assembled from GluN1/GluN3 subunits, and synaptic NMDARs in complexes with NMDAR-associated proteins remain elusive. Combining multiple techniques to analyze the structure, protein–protein interactions, function, and localization of NMDARs will contribute to a deeper understanding of NMDARs.

## Abbreviations

iGluRs: ionotropic glutamate receptors
NMDARs: *N*-methyl-d-aspartate receptors
AMPARs: α-amino-3-hydroxy-5-methyl-4-isoxazolepropionic acid receptors
KARs: kainate receptors
CNS: central nervous system
EPSC: excitatory postsynaptic current
cryo-EM: cryo-electron microscopy
ATD: amino-terminal domain
LBD: ligand-binding domain
TMD: transmembrane domain
CTD: C-terminal domain
PAMs and NAMs: positive and negative allosteric modulators
P_o_: open channel probability
PPDA: ((2S*,3R*)-1-(phenanthrene-2-carbonyl)piperazine-2,3-dicarboxylic acid
D-AP5: D-2-amino-5-phosphonopentanoate
NVP-AAM077: (R)-[(S)-1-(4-Bromo-phenyl)-ethylamino]-(2,3-dioxo-1,2,3,4-tetrahydroquinoxalin-5-yl)-methyl-phosphonic acid
ST3: {(*S*)-5-[(*R*)-2-amino-2-carboxyethyl]-1-[4-(3-fluoropropyl)phenyl]-4,5-dihydro-1*H*-pyrazole-3-carboxylic acid
SDZ220-040: (S)-α-Amino-2′,4′-dichloro-4-hydroxy-5-(phosphonomethyl)-[1,1′-biphenyl]-3-propanoic acid
L-689,560: *trans*-2-Carboxy-5,7-dichloro-4-phenylaminocarbonylamino-1,2,3,4-tetrahydroquinoline
PCP: phencyclidine
MD: molecular dynamics
smFRET: single-molecule fluorescence resonance energy transfer
EPR: electron paramagnetic resonance
